# First detection of Sindbis virus in wild birds in Nigeria

**DOI:** 10.1038/s41598-025-10556-3

**Published:** 2025-07-09

**Authors:** Dickson Anoibi Matthew, Edvin Karlsson, Jonathan Ajik Izang, Linn Isberg, Jonas Näslund, Andreas Sjödin, Ulf Ottosson, Olivia Wesula Lwande, Jonas Waldenström

**Affiliations:** 1 A. P. Leventis Ornithological Research Institute, University of Jos, Jos, Nigeria; 2 Swedish Defence Research Agency, CBRN, Defence and Security, Umeå, Sweden; 3 Department of Clinical Microbiology, Umeå University, 901 85 Umeå, Sweden; 4 Umeå Centre for Microbial Research (UCMR), Umeå University, Umeå, Sweden; 5 Centre for Ecology and Evolution in Microbial Model Systems, Linnaeus University, Kalmar, Sweden

**Keywords:** Sindbis virus (SINV), African Thrush, Wild birds, Arbovirus, Zoonosis, Nigeria, Alphaviruses, Animal migration, Pathogens

## Abstract

Sindbis virus (SINV) is a zoonotic arbovirus transmitted by mosquitoes and maintained by wild birds with an expanding distribution globally. Despite its importance, surveillance efforts are low or lacking in many areas, especially in Africa. Our study aimed to highlight the epidemiology of SINV in wild birds in a West African country – Nigeria – with implications for human health. Blood samples were collected from wild resident Afrotropical and migrant Palearctic birds over two years. RT-qPCR was used to detect SINV RNA positive samples, followed by confirmatory conventional PCR and Sanger sequencing targeting the non-structural protein gene. Three out of 504 samples (0.6%; 95% CI: 0.12–1.73%) were positive for SINV, all from individuals of a single species, the African Thrush (*Turdus pelios*). We successfully generated the whole genome sequence of one sample. Phylogenetic analysis revealed it was closely related to strains from Algeria, Spain and Kenya in the SINV-I genotype. The study suggests that SINV is enzootic in the region and that the African Thrush may be a putative reservoir species.

## Introduction

Sindbis virus (SINV) is an arthropod-borne Alphavirus (family Togaviridae) that causes a febrile illness in humans manifested by rash, arthralgia, fatigue, muscle pain, headache, itching, upper respiratory symptoms and fever^1^. It was first described from the Sindbis region of Egypt in 1952, when it was isolated from mosquitos in an area where patients presented with febrile illness, rash and arthritis^2^. A decade later it was detected in humans in South Africa^3,4^. Since then, the virus has been identified in other parts of the world, including Europe, Asia and Australia^1^. The disease caused by the virus is known by different names in different geographical areas, such as Pogosta disease in Finland, Karelian fever in Russia, and Ockelbo disease in Sweden^5^. Although a widespread virus, SINV disease burden has a disjunct distribution, with largest effects in South Africa and Scandinavia^5^.

SINV is zoonotic and is transmitted through the bite of infected mosquitoes, particularly species of the *Culex* genus^5,6^. Wild birds are the main reservoir hosts^5^, but the virus has been detected through RT-PCR in a range of wildlife (e.g. African Buffalo (*Syncerus caffer*), Sable antelope (*Hippotragus niger*), Giraffe (*Giraffa camelopardalis*), Blesbuck (*Damaliscus pygargus phillipsi*), and White rhinoceros (*Ceratotherium simum*)) and domestic vertebrates (sheep, pig, horse) that had neurological infection or unexplained deaths^7,8^. There is no known human-to-human transmission of the virus^9^. With wild birds being reservoirs, it is not surprising that they have been implicated in long-distance dispersal, possibly across continents, as the distribution of the virus reflects migratory bird pathways between the Northern and the Southern Hemisphere^10–12^.

SINV is grouped into six genotypes (SINV I-VI) based on the glycoprotein sequence of the E2 gene. The SINV-I genotype strains have been found in Europe, the Middle East and Africa; SINV-II in Australia and Malaysia; SINV-III in India and the Philippines; SINV-IV in Azerbaijan and China; SINV-V in New Zealand; and SINV-VI in south-west Australia^5,6^. The SINV genotype complex displays significant genetic variability both at the nucleotide level and the amino acid level^11,13^.

Despite the widespread detection of the virus, the actual disease burden and detailed knowledge of distribution of the virus and susceptible species is still limited. This is especially true in Africa due to under-reporting and limited surveillance efforts in many areas of the continent^12,14^. Compared to other regions of Africa, there is limited information about SINV from many parts of West Africa. This includes Nigeria, one of the largest and most populous countries in West Africa, where the only mention of SINV comes from a serosurvey of humans in 1978^15^. Furthermore, although many studies in Africa have reported detection and isolation of SINV in mosquitoes^1,6,15–17^, there is lack of information on the status of the virus in wild bird populations and how that influences the epidemiology of the virus in the region.

The ongoing geographic expansion of many arboviruses as a result of climate change is more and more in focus^18^. SINV has long been known to circulate in Southern and Eastern Africa but recently more infections have been detected outside the previously known distribution range^5^. However, to the best of our knowledge, there are no circulation records of SINV in either humans, vector mosquitoes, or host bird species in Nigeria, apart from the serology report of Fagbami^19^ when humans were seropositive to the virus in Southwest Nigeria. Our study therefore, aimed to highlight the epidemiology of SINV in wild birds in Nigeria, setting the stage for further investigation and surveillance. We carried out an intense field sampling project in central Nigeria and report for the first time detection of SINV in African Thrushes (*Turdus pelios*) and generally in wild birds in Nigeria.

## Materials and methods

### Ethical considerations

The experimental protocol was approved by the scientific committee of A.P. Leventis Ornithological Research Institute, University of Jos, and the work was carried out in accordance with institutional guidelines and national legislation. The methods are reported according to ARRIVE guidelines. A material transfer agreement on access to genetic resources was approved by the Nigeria Federal Ministry of Environment.

### Study design

**Fig. 1:**
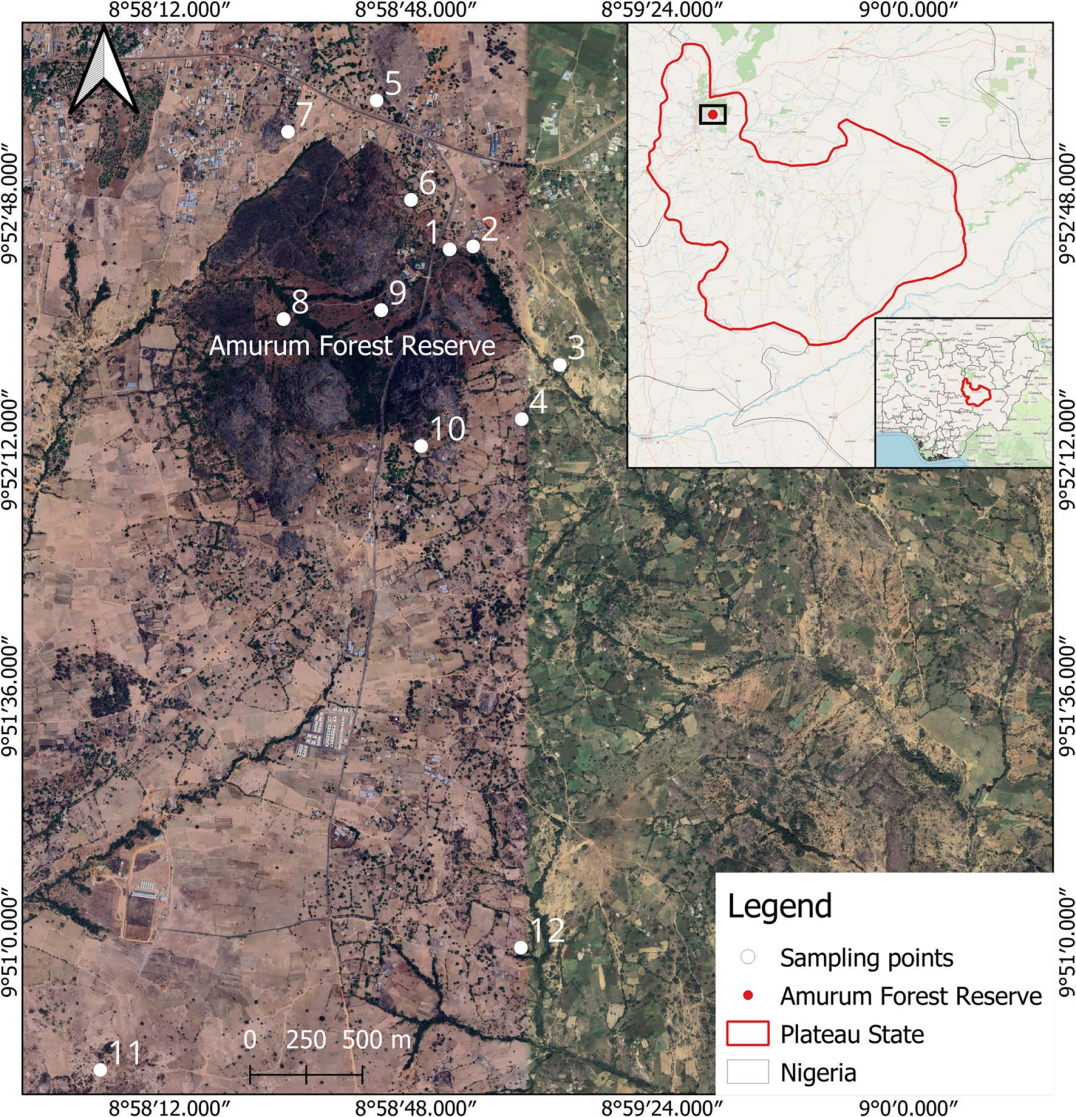
Study area map showing sampling points in Amurum Forest Reserve and surrounding areas. The map was created using QGIS (Version 3.28.3, https://qgis.org) and the basemap was sourced from Google Satellite via XYZ Tiles in QGIS and added as a QGIS map layer. Sampling sites were selected based on the observation of abundance of African Thrushes and Palearctic migrant species. The points in Sabon Gari were areas with open lands mixed with farmlands and interspersed with thickets, typical of where to find some Palearctic migrant species, e.g., Common Whitethroat (*Curruca communis*), Common Nightingale (*Luscinia megarhynchos*) and Whinchat (*Saxicola rubetra*). The other points included areas close to gallery forests and pools of water (especially in the dry season), thickets and covers, wooded lands and settlements, typical of where to find African Thrush in abundance. Keys: 1 = Eastern Gully 1, 2 = Eastern gully 2, 3 = Kerker 1, 4 = Kerker 2, 5 = Gidan Babawo 1, 6 = Gidan Babawo 2, 7 = Zarazon, 8 = Pythons’ gully, 9 = Zainab’s gully, 10 = Laminga village, 11 = Sabon Gari village 1, 12 = Sabon Gari village 2.

A cross-sectional study was carried out where birds were sampled every month for two years, 2022 and 2023. Fieldwork was conducted in Amurum Forest Reserve (09°53″ N, 08°59″ E) on the Jos Plateau, located 15 km northeast of the city of Jos, in Plateau State Nigeria (Fig. 1). Amurum Forest is about 300-ha large reserve comprising granitic outcrops in scrub Guinea savanna, interspersed with gallery forests and patches of grasslands. It is also the seat of the A.P. Leventis Ornithological Research Institute (APLORI)^20^. The Amurum Forest Reserve is an Important Bird Area with over 350 bird species recorded^20,21^, about 36% of the 966 birds occurring in Nigeria^22^. The reserve serves as wintering and stop-over site for many Palearctic migratory bird species, as well as breeding grounds for resident and intra-African migratory birds^23^. The diversity of avian assemblages and the close proximity to human settlement that makes it suitable for zoonotic diseases transmission, as well as the presence of potential SINV vectors in the area^24,25^ informed the choice of the reserve as the study site. Temperature in this area ranges from 8˚C to 38˚C, and mean annual rainfall is 1411mm^26^. Rainfall varies across the months of the year, with the rainy season lasting for 7 months (April–October) and dry season lasting for 5 months (November–March)^27,28^. Wild birds were captured weekly using mist nets, and ringed, with appropriate morphometrics recorded^29^. An average of 10 mist nets of various sizes (9, 12, and 18 m long) were used to capture birds at 12 different locations within and around the reserve (Fig. 1). Nets were opened early in the morning while still dark (between 5:00- 5:30 AM) and bird ringing and blood sampling continued until 11:00 AM^29^.

Blood samples were collected from the brachial veins of birds using 25G needles^29,30^ and 70µL heparinised micro hematocrit tubes. The target groups were thrushes, robin-chats and chats and Palearctic migratory species, but few samples were collected from other species (Table 1), when they were caught in the nets. The reason for targeting particular species, especially the African Thrush was that thrushes are generally considered as an important amplifying host of SINV, at least in Europe. In Sweden, prevalence of neutralizing antibodies in many thrush species reached 70%^31^^.^ In other European studies, thrushes recorded high antibody prevalence and many thrush species were found competent for SINV and were potentially able to infect mosquito vectors with the virus, ensuring continuous viral circulation^32,33^. This role of being an important host for SINV by thrushes is likely the same in Africa as SINV antibody prevalence reached 94% in Olive Thrush in South Africa^4^. Given these scenarios and the lack of information on SINV occurrence in wild birds in Nigeria, thrushes would be the choice candidate for investigating SINV in wild birds in the area. In addition, the African Thrush is the most common and widespread of the thrushes in this region. Furthermore, its synanthropic nature that enables interaction with humans and animals, its ground feeding behaviour, and preference for thickets and covers that likely predisposes it to mosquito bite all made it our preferred species to start investigating arboviruses, including SINV in birds.

**Table 1:** Names of birds and the number of each species sampled (decreasing order) including the number positive for SINV through RT-qPCR screening.

Species	Scientific name	Origin	Number sampled	Number positive
African Thrush	*Turdus pelios*	Afrotropical	378	3
Snowy-crowned Robin-chat	*Cossypha niveicapilla*	Afrotropical	42	0
Laughing Dove	*Spilopelia senegalensis*	Afrotropical	10	0
Bronze-tailed Starling	*Lamprotornis chalcurus*	Afrotropical	7	0
Garden Warbler	*Sylvia borin*	Palearctic migrant	7	0
Familiar Chat	*Oenanthe familiaris*	Afrotropical	6	0
Village Weaver	*Ploceus cucullatus*	Afrotropical	6	0
Whinchat	*Saxicola rubetra*	Palearctic migrant	6	0
Brown Babbler	*Turdoides plebejus*	Afrotropical	5	0
Black-crowned Tchagra	*Tchagra senegalus*	Afrotropical	4	0
White-crowned Cliff Chat	*Thamnolaea coronata*	Afrotropical	4	0
Common Whitethroat	*Curruca communis*	Palearctic migrant	4	0
Green Wood Hoopoe	*Phoeniculus purpureus*	Afrotropical	3	0
Eurasian Wryneck	*Jynx torquilla*	Palearctic migrant	3	0
African Moustached Grass-warbler	*Melocichla mentalis*	Afrotropical	2	0
Eurasian Blackcap	*Sylvia atricapilla*	Palearctic migrant	2	0
Common Rock Thrush	*Monticola saxatilis*	Palearctic migrant	2	0
Common Nightingale	*Luscinia megarhynchos*	Palearctic migrant	2	0
Blackcap Babbler	*Turdoides reinwardtii*	Afrotropical	1	0
Chestnut-crowned Sparrow Weaver	*Plocepasser superciliosus*	Afrotropical	1	0
African Hoopoe	*Upupa africana*	Afrotropical	1	0
Long-tailed Nightjar	*Caprimulgus climacurus*	Afrotropical	1	0
Northern Black Flycatcher	*Melaenornis edolioides*	Afrotropical	1	0
Pale Flycatcher	*Agricola pallidus*	Afrotropical	1	0
Common Redstart	*Phoenicurus phoenicurus*	Palearctic migrant	1	0
Speckled Mousebird	*Colius striatus*	Afrotropical	1	0
Tree Pipit	*Anthus trivialis*	Palearctic migrant	1	0
Yellow-billed Shrike	*Lanius corvinus*	Afrotropical	1	0
Yellow-gorgeted Greenbul	*Atimastillas flavicollis*	Afrotropical	1	0

The blood was transferred to Type I Advantec® (Tokyo, Japan) Nobuto blood sampling filter paper and absorbed as dry blood spots^34,35^ which were placed in labelled Whirl–Pak® Standard Sterilized bags after drying in the field under shade. The bags with the paper strips were then kept in a cooler box until transported to the APLORI molecular ecology lab where they were kept at −20℃ until screening. The samples were shipped on dry ice from Nigeria to the Department of Clinical Microbiology, Umeå University, Sweden for processing. Each dry blood on Nobuto filter paper strip was eluted in 1 mL of phosphate-buffered saline (PBS) containing 0.05% Tween and 0.08% sodium azide in a 2 mL screw-capped tube on a shaking rack at 4℃ for a minimum of 12 h before RNA extraction.

### RNA extraction and RT-qPCR screening

Viral RNA was extracted using a QIAamp Viral RNA Mini Kit (Qiagen, Hilden, Germany) according to the manufacturer’s instructions. A 140 μL eluted Dry Blood Spot (DBS) sample volume was used and RNA was eluted in a final volume of 60 μL, collected in a 1.5 mL sterile microcentrifuge tube and stored at − 80 ℃.

SINV RNA was detected using RT-qPCR on a QuantStudio™ 5 System thermocycler (ThermoFisher Scientific, Waltham, USA) using Biosystems qPCRBIO Probe 1-step Go Lo-ROX kit at optimized cycling conditions: 1 cycle at 50 °C for 10 min, 1 cycle at 95 °C for 2 min; 40 cycles at 95 °C for 5 s and 60 °C for 25 s with primers targeting the nonstructural gene one (nsP1); SINV-nsP1F, 5′-GGTTCCTACCACAGCGACGAT-3′, SINV-nsP1R, 5′-TGATACTGGTGCTCGGAAAACA-3′, and SINV-nsP1 probe, 5′-FAM-TTGGACATAGGCAGC GCA-3′^36^. The final reaction volume was 20 µL, containing 5 µL of template RNA. The SINV Lövånger strain (Accession number: KF737350) was used as a positive control and nuclease free water was used as a negative control in the RT-qPCR.

### Hybridization capture and Illumina sequencing

To confirm the RT-qPCR screening, we selected SINV positive samples for whole genome sequencing using the Twist Comprehensive Viral Research Panel (CVRP) (Twist Biosciences, San Francisco, USA). In brief, Illumina TruSeq-compatible libraries were generated using the Twist Library Preparation EF Kit 2.0 with Enzymatic Fragmentation and the Twist Universal Adapter System following the manufacturer’s protocol. Hybridization capture was performed on the generated library using the CVRP and the Twist Target Enrichment Standard Hybridization v1 workflow. The indexed sample library was approximately 8.62 ng/μL and was used in a multiplexed 16-h hybridization capture reaction. Following enrichment, the library was sequenced with 150 bp paired-end reads on the MiSeq platform (Illumina, CA, USA), using the MiSeq Reagent Nano Kit, v2 300-cycle kit. Generated sequenced reads were assembled using Megahit^37^ and both reads and assemblies were classified using kraken2^38^. The data processing was performed using the workflow manager Snakemake^39^ together with Bioconda^40^.

### cDNA synthesis, conventional PCR, gel electrophoresis and Sanger sequencing

The enrichment experiments only gave a partial sequence of SINV genome from one of the three RT-qPCR positive samples. We therefore, subjected the three samples to conventional PCR for confirmation of bands on gels and further purification for Sanger sequencing of the non-structural protein (nsp4) gene. The positive RNA samples were converted to cDNA using Thermo Scientific RevertAid RT Kit (Thermo Fisher Scientific, Waltham, USA) using random primers, according to the manufacturer’s instructions. The cDNA synthesis conditions were: first incubation at 25 °C for 5 min, second incubation at 42 °C for 1 h and a termination step at 70 °C for 5 min. The conventional PCR was carried out using forward primer SINV1 (5′-TTTAGCGGATCGGACAATTC-3′) and reverse primer SINV2 (5′-GCGGTGACGAACTCAGTAG-3′) targeting the nsp4 gene of the virus^15^, and Thermo Fisher Scientific’s Phusion Green Hot Start II High-Fidelity PCR Master Mix. The SINV Lövånger strain (Accession number: KF737350) was used as a positive control and nuclease free water was used as a negative control just like the RT-qPCR. The final reaction volume was 20 µL containing 5 µL of template cDNA. The cycling conditions included a first step of initial denaturation (one cycle) at 98 °C for 30 s, a second step of 35 cycles of Denaturation at 98 °C for 7 s, annealing at 52 °C for 15 s, and extension at 72 °C for 20 s; with a third step of final extension at 72 °C for 7 min for 1 cycle. The PCR products were visualized using gel electrophoresis (1.2% agarose in 1 × TAE with GelRed (Biotium Inc. Hayward, CA, US). Positive samples were purified with ExoSAP-IT kit (Thermo Fisher Scientific) and sequenced using Sanger sequencing by Eurofin Genomics (Germany). Nucleotide sequences were then aligned to available SINV sequences in GenBank using the Basic Local Alignment Search Tool (BLAST) of National Center for Biotechnology Information.

### Tiling PCR and nanopore sequencing

After confirming the presence of SINV through Sanger sequencing, we designed a tiled sequencing primer panel based on publicly available SINV genomes to increase the likelihood of generating full genome sequences from the three RT-qPCR positive samples. Complete SINV genomes were downloaded from the BV-BRC public repository on November 12, 2024^41^. We excluded duplicate genome sequences and experimentally evolved SINV lineages. Subsequently, we aligned SINV genome sequences belonging to genotype I (n = 87, excluding MF409177) using MAFFT (v7.508)^42^ with the auto setting (see supplementary table S1 for full list of included accession numbers). The resulting multiple sequence alignment was then used as input for varVAMP (v1.2.1)^43^ to generate tiled sequencing primers, with the following parameters: consensus nucleotide threshold of 1, maximum ambiguous nucleotides per primer set to 2, optimal amplicon length of 1200 bp, maximum amplicon length of 1500 bp, and an overlap of 200 bp between amplicons.

We further evaluated primer specificity by mapping the primers and the Illumina reads generated during enrichment to the closest matching SINV reference strain available at the time (OK644705), identified using Mash Screen (v2.3)^44^. Mapping was performed with Minimap2 (v2.24-r1122)^45,46^ and SAMtools (v1.16.1)^47^, and visualized in IGV^48^. We manually corrected potential mismatches between the mapped reads and primer sequences by introducing additional ambiguous nucleotides into the primers (See supplementary table S2 for primer sequences). Additionally, we designed primer SINV_10_RIGHT_EXT to improve genome coverage. We ordered all primers from Eurofins Genomics (Ebersberg, Germany) and combined them into two separate equimolar 10 µM non-overlapping pools (Pool 1 and Pool 2, Supplementary table S2).

The tiled amplicon sequencing protocol was adapted from Quick et al.^49^, with a few differences. We used RNA from the SINV positive samples, SINV strain Lövånger (KF737350, positive control), and nuclease free water (negative control) and reverse-transcribed them into cDNA using ProtoScript II first strand synthesis kit (New England Biolabs, Ipswich, MA, USA), following the manufacturer’s protocol. A volume of 2.5 µl cDNA was used as template for the tiling PCR, which was conducted in two separate reactions with 600 nM of primer pool 1 and 500 nM of primer pool 2, respectively, in a total volume of 25 µl 1X Q5 Hot Start High-Fidelity Master Mix (New England Biolabs). The thermal cycling conditions were as follows: 98 °C for 30 s, followed by 40 cycles at 98 °C for 15 s, 63 °C for 30 s, 72 °C for 90 s, with a final extension at 72 °C for 2 min. Samples were held at 4 °C until purified using 1X Ampure XP beads (Beckman-Coulter, Brea, CA, USA) according to manufacturer’s protocol. Only the sample that had previously generated sequence data during the enrichment experiment produced amplicons and was subsequently advanced, along with the controls, for sequencing.

We combined and prepared equal amounts of amplicons from pool 1 and pool 2 for sequencing using Native Barcoding Kit 24 V14 (SQK-NBD114.24, Oxford Nanopore Technologies, Oxford, UK), as per the protocol. The libraries were sequenced on a MinION flow cell (FLO-MIN114). Primer pair SINV_10_LEFT and SINV_10_RIGHT_EXT, along with primer pairs that showed poor tiling coverage when pooled, were used in singleplex PCRs and resequenced alongside the PCR amplicons generated by the two primer pools.

We performed basecalling and demultiplexing using Dorado (v0.9.0 + 9dc15a8; Oxford Nanopore Technologies) with super accuracy (sup). We filtered demultiplexed reads misplaced due to barcode bleeding by mapping the reads to OK644705 and KF737350 using minimap2. We then generated consensus sequences from the remaining reads using Artic (v1.5.7; https://github.com/artic-network/fieldbioinformatics) with reference genome KF737350 and basecalling model r1041_e82_400bp_sup_v500.

### Phylogenetic analysis

We aligned the consensus sequences of the whole genome generated from tiled amplicon sequencing with publicly available complete SINV genomes (BV-BRC, January 23, 2025) and the Whataroa virus genome (NC_016961.1) using MAFFT (n = 114, Supplementary table S1). A maximum likelihood tree was constructed using IQ-TREE (v2.3.6)^50^ with ModelFinder^51^, 1000 ultrafast bootstrap replicates^52^, and NC_016961.1 as the outgroup. We visualized the phylogenetic tree using iTol (v7)^53^.

## Results

We screened dry blood spots from 504 birds of 29 species (20 Afrotropical species and 9 Palearctic migratory species; Table 1 and 2). A total of 3 (0.6%; 95% CI: 0.12–1.73%) were positive for SINV from RT-qPCR screening (Table 1 and 2). The cycle threshold (Ct) values for the three positive samples were 28.47, 37.35 and 38.55, respectively. All three positive samples were collected from adult African Thrushes (*Turdus pelios*).

**Table 2:** Distribution of sampled birds across sampling locations.

Location	Site	Common name	Scientific name	Sampled	Positive
Amurum forest	Eastern Gully 1	African Thrush	*Turdus pelios*	43	2
Amurum forest	Eastern Gully 1	Eurasian Wryneck	*Jynx torquilla*	1	
Amurum forest	Eastern Gully 1	Brown Babbler	*Turdoides plebejus*	1	
Amurum forest	Eastern Gully 1	Bronze-tailed Starling	*Lamprotornis chalcurus*	1	
Amurum forest	Eastern Gully 1	Village Weaver	*Ploceus cucullatus*	1	
Amurum forest	Eastern Gully 1	Black-crowned Tchagra	*Tchagra senegalus*	1	
Amurum forest	Eastern Gully 1	African Moustached Grass-warbler	*Melocichla mentalis*	2	
Amurum forest	Eastern Gully 1	Speckled Mousebird	*Colius striatus*	1	
Amurum forest	Eastern Gully 2	Common Redstart	*Phoenicurus phoenicurus*	1	
Amurum forest	Eastern Gully 2	African Thrush	*Turdus pelios*	37	
Amurum forest	Eastern Gully 2	Snowy-crowned Robin-chat	*Cossypha niveicapilla*	15	
Amurum forest	Eastern Gully 2	Whinchat	*Saxicola rubetra*	1	
Amurum forest	Eastern Gully 2	Village Weaver	*Ploceus cucullatus*	1	
Amurum forest	Eastern Gully 2	Laughing Dove	*Spilopelia senegalensis*	3	
Amurum forest	Eastern Gully 2	Garden Warbler	*Sylvia borin*	2	
Amurum forest	Eastern Gully 2	Eurasian Blackcap	*Sylvia atricapilla*	2	
Amurum forest	Eastern Gully 2	Blackcap Babbler	*Turdoides reinwardtii*	1	
Amurum forest	Python’s Gully	African Thrush	*Turdus pelios*	19	
Amurum forest	Python’s Gully	Bronze-tailed Starling	*Lamprotornis chalcurus*	2	
Amurum forest	Zainab’s Gully	African Thrush	*Turdus pelios*	21	1
Amurum forest	Zainab’s Gully	Laughing Dove	*Spilopelia senegalensis*	1	
Amurum forest	Zainab’s Gully	Common Rock Thrush	*Monticola saxatilis*	1	
Gidan Babawo	Babawo1	African Thrush	*Turdus pelios*	21	
Gidan Babawo	Babawo1	Snowy-crowned Robin-chat	*Cossypha niveicapilla*	4	
Gidan Babawo	Babawo1	Long-tailed Nightjar	*Caprimulgus climacurus*	1	
Gidan Babawo	Babawo 2	African Thrush	*Turdus pelios*	25	
Gidan Babawo	Babawo 2	Brown Babbler	*Turdoides plebejus*	3	
Kerker	Kerker 1	African Thrush	*Turdus pelios*	19	
Kerker	Kerker 1	Snowy-crowned Robin-chat	*Cossypha niveicapilla*	4	
Kerker	Kerker 1	Village Weaver	*Ploceus cucullatus*	1	
Kerker	Kerker 1	Common Whitethroat	*Curruca communis*	1	
Kerker	Kerker 1	Bronze-tailed Starling	*Lamprotornis chalcurus*	4	
Kerker	Kerker 1	Eurasian Wryneck	*Jynx torquilla*	2	
Kerker	Kerker 2	African Thrush	*Turdus pelios*	41	
Kerker	Kerker 2	Familiar Chat	*Oenanthe familiaris*	2	
Kerker	Kerker 2	Yellow-billed Shrike	*Lanius corvinus*	1	
Kerker	Kerker 2	African Hoopoe	*Upupa africana*	1	
Kerker	Kerker 2	Yellow-gorgeted Greenbul	*Atimastillas flavicollis*	1	
Laminga	Laminga	Village Weaver	*Ploceus cucullatus*	1	
Laminga	Laminga	African Thrush	*Turdus pelios*	24	
Laminga	Laminga	White-crowned Cliff Chat	*Thamnolaea coronata*	1	
Laminga	Laminga	Familiar Chat	*Oenanthe familiaris*	3	
Laminga	Laminga	Garden Warbler	*Sylvia borin*	3	
Laminga	Laminga	Laughing Dove	*Spilopelia senegalensis*	6	
Sabon Gari	Sabon Gari 1	African Thrush	*Turdus pelios*	10	
Sabon Gari	Sabon Gari 1	Common Rock Thrush	*Monticola saxatilis*	1	
Sabon Gari	Sabon Gari 1	Pale Flycatcher	*Agricola pallidus*	1	
Sabon Gari	Sabon Gari 1	Whinchat	*Saxicola rubetra*	1	
Sabon Gari	Sabon Gari 1	Familiar Chat	*Oenanthe familiaris*	1	
Sabon Gari	Sabon Gari 1	Snowy-crowned Robin-chat	*Cossypha niveicapilla*	3	
Sabon Gari	Sabon Gari 1	Common Whitethroat	*Curruca communis*	3	
Sabon Gari	Sabon Gari 2	African Thrush	*Turdus pelios*	58	
Sabon Gari	Sabon Gari 2	Snowy-crowned Robin-chat	*Cossypha niveicapilla*	12	
Sabon Gari	Sabon Gari 2	Chestnut-crowned Sparrow Weaver	*Plocepasser superciliosus*	1	
Sabon Gari	Sabon Gari 2	Garden Warbler	*Sylvia borin*	2	
Sabon Gari	Sabon Gari 2	Green Wood Hoopoe	*Phoeniculus purpureus*	3	
Sabon Gari	Sabon Gari 2	Common Nightingale	*Luscinia megarhynchos*	2	
Sabon Gari	Sabon Gari 2	Whinchat	*Saxicola rubetra*	1	
Zarazon	Zarazon	African Thrush	*Turdus pelios*	60	
Zarazon	Zarazon	Whinchat	*Saxicola rubetra*	3	
Zarazon	Zarazon	Tree Pipit	*Anthus trivialis*	1	
Zarazon	Zarazon	White-crowned Cliff Chat	*Thamnolaea coronata*	3	
Zarazon	Zarazon	Snowy-crowned Robin-chat	*Cossypha niveicapilla*	4	
Zarazon	Zarazon	Brown Babbler	*Turdoides plebejus*	1	
Zarazon	Zarazon	Black-crowned Tchagra	*Tchagra senegalus*	3	
Zarazon	Zarazon	Village Weaver	*Ploceus cucullatus*	2	
Zarazon	Zarazon	Northern Black Flycatcher	*Melaenornis edolioides*	1	
Total				**504**	**3**

**Fig. 2:**
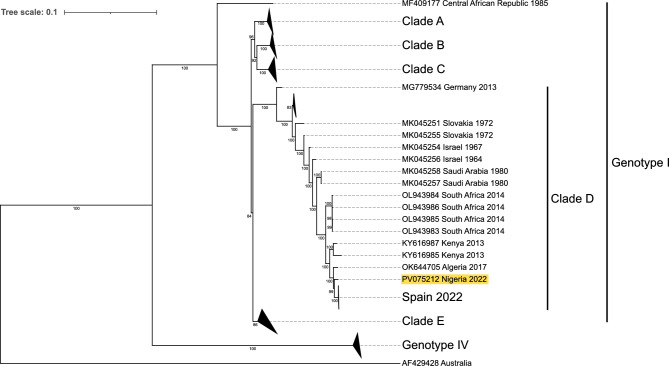
A maximum likelihood tree constructed from complete SINV genomes. Genome AF429428 is shown as the root, with major clades collapsed (except Clade D). The Whataroa virus genome (NC_016961.1) was used as the outgroup but is not shown.

Conventional PCR confirmed two out of the three RT-qPCR positive samples but only one sample gave clear band to use for sequencing. Sanger sequence of SINV nsp4 gene from the sample that produced a clear and sharp band on gel (this had the lowest cycle threshold value [Ct = 28.47] from RT-qPCR) has been deposited in GenBank (Accession number: PQ666553). Result of BLAST revealed its close identity with SINV strains of genotype I from Kenya, Algeria and Spain with 98.69%, 98.60% and 98.60% identities respectively. Tiled amplicon sequencing yielded the whole genome sequence for one of the three RT-qPCR positive samples (the one that showed sharp band on gel and had the lowest Ct value with RT-qPCR). The length of the genome was 11,532 bp. The result of phylogenetic analysis of this whole genome sequence revealed that this isolate was in clade D of SINV genotype 1 group and clustered with Kenyan, Algerian and Spanish sequences of mosquito isolates (Fig. 2), just like the nsp4 gene sequence.

## Discussion

Knowledge about SINV in West Africa is limited compared to other regions in Africa. Our study provides the first evidence of SINV occurrence in wild birds in Nigeria and the broader region of West Africa. This finding highlights the need for improved arbovirus surveillance targeting a range of potential hosts in the region, as well as investigations into the epidemiology of SINV and other arboviruses and their ecological drivers. Although the prevalence of SINV was low in this study, detecting viral RNA indicates active circulation of the virus. However, it is worthy of note that the period of viremia of SINV is short, typically between 2–5 days^31^. As such it may be difficult to track the true extent of its occurrence in the bird population. A broadened approach would be to combine virus detection from reservoir hosts with vector screening, as well as the use of serology to explore the history of infection and get an idea of how widespread the virus is in bird and human populations. Unfortunately, we did not sample mosquitoes in this study, but the detection of SINV in avian blood samples implies the likely presence of the vectors in the area. Moreover, other studies that involved mosquito sampling have reported potential SINV vectors such as *Culex quinquefasciatus* to be abundant in the nearby city of Jos^24,25^. Therefore, sampling and characterizing mosquito populations in this area should be a priority in future studies.

Given the under-reporting of SINV in Africa in general and West Africa in particular, we cannot apriori know what is expected regarding the epidemiological status of the virus. An evident first question to ask is whether the detection of SINV here is the result of spillover infections introduced by wintering Palearctic birds that may be coming from SINV endemic regions in Europe, or that SINV is endemic, but undetected in the study area. To start with, it is interesting to note that the only species positive for the virus was the African Thrush, a common resident and intra-African migratory species that occurs in sub-Saharan Africa. This species is from the same genus of thrushes (*Turdus*) that in previous studies have been identified as important amplifying hosts of SINV in Europe and Africa^4,31,54^. Hence, biologically the African Thrush could potentially serve a similar role in Africa. At Amurum, African Thrushes are present year-round, but seeing an influx of individuals during the months of February-April is likely a result of intra-African migration of birds heading for breeding areas in the Sudan and Sahel savanna. We did not detect SINV RNA in any of the 28 individuals of 9 Palearctic migratory bird species that were sampled. The sample of each species (average of 3 samples per species) is too small to draw firm conclusion on their role as source of SINV in the area. One can note, however, that SINV-positive samples were all from the dry season, with detections in March which coincides with the period of influx of the African Thrush. Palearctic migrants arrive in Amurum in the autumn, usually in late August/early September, and consists of a pulse of species aiming for wintering areas further south, and another set of species, such as Common Whitethroat (*Curruca communis*), Garden Warbler (*Sylvia borin*), Tree Pipit (*Anthus trivialis*) and Eurasian Wryneck (*Jynx torquilla*) that winter in the Amurum area. Given that SINV is an acute viral infection, requiring an arthropod vector for transmission, detections in March means that if Palearctic birds are responsible for a recent introduction, it would still have required multiple rounds of bird-vector infections to be maintained until our detections in African Thrushes. Moreover, passing the Mediterranean Sea and the Saharan desert is physiologically taxing, and if infection lowers the bird’s condition even slightly the risk of succumbing during migration is high^55^. Another factor to consider is the speed of migration. Long-distance Palaearctic passerine migrants generally adopt a migration strategy with several legs of migration, interspersed with stays in stopover sites where fat stores are replenished. This means that it can take more than a month and several stopovers for a passerine bird to reach the wintering ground^56^, and given the short viremic period (2–5 days) of SINV^31^, viremia may wane significantly before the bird reaches the African wintering ground. Considering these facts, we hypothesize that SINV is more likely an undetected enzootic infection in the studied avifauna. Given the zoonotic potential of the virus, its presence in African Thrushes represents a public health concern as the bird is associated with human settlement. Moreover, previous studies have linked SINV outbreaks and increased seroprevalence in human to spatial and temporal fluctuation in the population of wild birds^32,54^. Particularly, Lundström et al.^54^ reported that areas with higher densities of birds corresponded with increased human cases of SINV infection in Sweden. Experimental infection of birds with SINV revealed that thrushes were highly competent, developing viremia sufficient to infect mosquitoes^31,33^. Their African counterpart, the African Thrush as well as other species may have similar status, thereby maintaining the virus in the local area. Therefore, there is need for a more widespread sampling effort not only in birds, but also in mosquitoes, as well as an awareness among public health officials that SINV is a putative infection in the human population.

Phylogenetic analysis of the nsP4 gene, and the whole genome showed a close relationship between the African Thrush SINV sequence and mosquito strains from Algeria, Kenya and Spain^1,57,58^ all of which are members of the genotype 1 (SINV-I) known to circulate in Europe and Africa causing serious disease burden in humans^5^. The similarity of our strain to strains from other African locales supports the presence of more widely distributed African SINV strains rather than introduced European lineages. Furthermore, the closely related Spanish strain has been suggested to originate in Africa in relatively recent time^58^. Therefore, one can hypothesize that SINV distribution could be driven by seasonal intra-African bird migration, facilitating its movement across subregions. Migratory birds have been implicated in SINV spread within and across continents, including the supposed first transmission from Africa to Europe^1,6,11^. However, like other bird-borne arboviruses, the ecological dynamics of SINV in endemic settings are influenced by the assemblage of bird species, vector populations, and seasonal variations in both host and vector abundance^5,54^, factors that as of yet are poorly studied in our study area.

Regarding vectors and seasonality of the virus detection in this study, it is interesting to note the time of virus detection was in the dry season, when water is scarce and streams are reduced to pools. Such water-scarce condition can foster concentration of birds and mosquitoes around limited water bodies, thereby potentially increasing virus transmission in local patches. Evidently, more research is needed to characterize ecology and epidemiology in this system.

## Conclusion

Here for the first time, we confirm the presence of the SINV in African Thrush in Nigeria and the broader West Africa, highlighting the potential role of wild birds in its local transmission. Because mosquitoes are the principal vectors of SINV, detecting the virus in birds indicates an active vector-borne cycle. We recommend adopting a One Health surveillance framework that integrates wildlife (avian), vector (mosquito), and human sampling. This coordinated, multi-sectorial approach will help clarify the SINV ecology, support early detection of human infection, and guide effective prevention and control strategies for both wildlife and public health.

## Supplementary Information

Supplementary Information 1.

## Data Availability

Sequences of the nsp4 gene as well as the whole genome are deposited in Genbank with accession numbers PQ666553 and PV075212, respectively. All other data is found in the article or in the electronic supplementary files.
