# Supplementary material for: First detection of Sindbis virus in wild birds in Nigeria
Source: Sci Rep. 2025 Jul 9;15:24621. doi: 10.1038/s41598-025-10556-3 (PMC12241308; doi:10.1038/s41598-025-10556-3)
Supplement: Supplementary file 1 — Supplementary Information 1. [file 41598_2025_10556_MOESM1_ESM.pdf]

Supplementary information to

First detection of Sindbis virus in wild birds in Nigeria

Dickson Anoibi Matthew[1], Edvin Karlsson[2], Jonathan Ajik Izang[1], Linn Isberg[4], Jonas Näslund[2], Andreas Sjödin[2], Ulf Ottosson[1], Olivia Wesula Lwande[3,4*], Jonas Waldenström[5*]

1. A. P. Leventis Ornithological Research Institute P.O. Box 13404, Jos, Nigeria
2. Swedish Defence Research Agency, CBRN, Defence and Security, Umeå, Sweden
3. Department of Clinical Microbiology, Umeå University, 901 85, Umeå, Sweden
4. Umeå Centre for Microbial Research (UCMR), Umeå University, Umeå, Sweden
5. Centre for Ecology and Evolution in Microbial Model Systems, Linnaeus University, Kalmar, Sweden

*** Correspondence authors:**
Olivia Wesula Lwande Email: olivia.lwande@umu.se
Jonas Waldenström Email: jonas.waldenstrom@lnu.se

Supplementary Table S1. Accession numbers of Sindbis virus genome sequences used for designing the tiled sequencing primers (Tiling panel, n = 87), as well as the sequences used for multiple alignment and phylogenetic tree construction (Tree, n = 114).

Accession	Genome name	Isolation country	Isolation year	Tiling panel	Tree
PV075212	This study	Amurum Forest Nigeria	2022		x
AF103728	Sindbis virus XJ-160	China			x
AF103734	Sindbis-like virus YN87448			x	x
AF429428	Sindbis virus SW6562	Australia			x
BD269910	Sindbis virus			x	
BD269911	Sindbis virus			x	
GU361116	Sindbis virus 5.3	Germany	2009	x	x
GU361118	Sindbis virus 28.9	Germany	2009	x	x
HM147984	Babanki virus			x	x
JQ771793	Sindbis virus Ilomantsi-2005M	Finland	2005	x	x
JQ771794	Sindbis virus Ilomantsi-2002A	Finland	2002	x	x
JQ771795	Sindbis virus Ilomantsi-2002B	Finland	2002	x	x
JQ771796	Sindbis virus Ilomantsi-2002C	Finland	2002	x	x
JQ771797	Sindbis virus Johannes-2002	Finland	2002	x	x
JQ771798	Sindbis virus Kiihtelysvaara-2002	Finland	2002	x	x
JQ771799	Sindbis virus LEIV-9298	Russia	1983	x	x
JX570540	Sindbis virus Berlin-2010A	Germany	2012	x	x
KF737350	Sindbis virus Lovanger	Sweden	2013	x	x
KF981618	Kyzylagach virus LEIV-65A	Azerbaijan	1969		x
KY616984	Sindbis virus BUD_12001_KENYA_2010	Kenya	2010	x	x
KY616985	Sindbis virus BONI_584_KENYA_2013	Kenya	2013	x	x
KY616986	Sindbis virus KSM_1008_KENYA_2007	Kenya	2007	x	x
KY616987	Sindbis virus BONI_566_KENYA_2013	Kenya	2013	x	x
KY616988	Sindbis virus NVS_305_KENYA_2007	Kenya	2007	x	x
M69205	Ockelbo virus Edsbyn 82-5	Sweden		x	x

MF409177	Sindbis virus ArB489	Central African Republic	1985		x
MF409178	Babanki virus HB741570	Central African Republic	1974	x	x
MF459683	Sindbis virus Girdwood			x	x
MF543016	Sindbis virus Giessen_2016-A	Germany	2016	x	x
MF589985	Sindbis virus BNI-10865	Germany	2016	x	x
MG182396	Sindbis virus 18953	Australia	1975		x
MG495620	Sindbis virus BNI-CuliMo543	Germany	2016	x	x
MG679373	Sindbis virus Altay	Russia		x	x
MG679374	Sindbis virus Kyzylagach LEIV-65A	Azerbaijan	1963		x
MG679375	Sindbis virus Stavropol	Russia			x
MG679376	Sindbis virus F-720	Armenia	1990	x	x
MG679377	Sindbis virus LEIV-Ast03-1-839	Russia	2003	x	x
MG679378	Sindbis virus LEIV-Ast03-1-844	Russia	2003	x	x
MG679379	Sindbis virus SV-1383	Russia		x	x
MG679380	Sindbis virus Tatarstan	Russia		x	x
MG679381	Sindbis virus LEIV-9298	Russia		x	x
MG779533	Sindbis virus 15Z03121	Germany	2015	x	x
MG779534	Sindbis virus H7	Germany	2013	x	x
MG779535	Sindbis virus Z158523	Germany	2016	x	x
MH212167	Sindbis virus ArB7761	Central African Republic	1977	x	x
MH229928	Sindbis virus YN_222	China	2013		x
MK045224	Sindbis virus E597/Ae_rossicus/Sweden/2002	Sweden	2002	x	x
MK045225	Sindbis virus E594/Ae_rossicus/Sweden/2002	Sweden	2002	x	x
MK045226	Sindbis virus 84M140/Culi_morsitans_Edsbyn/Sweden/1984	Sweden	1984	x	x
MK045227	Sindbis virus 83M108/Cx_pipiens_torrentium/Sweden/1983	Sweden	1983	x	x
MK045228	Sindbis virus 83M107/Culi_morsitans/Sweden/1983	Sweden	1983	x	x
MK045229	Sindbis virus 92/Ae_sp/Norway/1983	Norway	1983	x	x
MK045230	Sindbis virus 86828/Culi_morsitans/Sweden/1985	Sweden	1985	x	x
MK045231	Sindbis virus 95M116/A_cinereus/Sweden/1995	Sweden	1995	x	x
MK045232	Sindbis virus 86752/Cx_pipiens_torrentium/Sweden/1985	Sweden	1985	x	x

MK045233	Sindbis virus 85M78/Aedes_cinereus_Edsbyn/Sweden/1985	Sweden	1985	x	x
MK045234	Sindbis virus 85M94/Cx_pipiens_torrentium/Sweden/1985	Sweden	1985	x	x
MK045235	Sindbis virus E917/Cx_pipiens_torrentium/Sweden/2002	Sweden	2002	x	x
MK045236	Sindbis virus 85M68/Culi_morsitans/Sweden/1985	Sweden	1985	x	x
MK045237	Sindbis virus 86520P3/Cx_pipiens_torrentium/Sweden/85	Sweden	1985	x	x
MK045238	Sindbis virus E945/Aedes_rossicus/Sweden/2002	Sweden	2002	x	x
MK045239	Sindbis virus 09_M_991/Cx_torrentium/Sweden/2009	Sweden	2009	x	x
MK045240	Sindbis virus F231/Ae_cinereus/Sweden/2003	Sweden	2003	x	x
MK045241	Sindbis virus 09_M_519/Cx_pipiens/Sweden/2009	Sweden	2009	x	x
MK045242	Sindbis virus 09_M_564/Cx_torrentium/Sweden/2009	Sweden	2009	x	x
MK045243	Sindbis virus 09_M_526/Cx_torrentium/Sweden/2009	Sweden	2009	x	x
MK045245	Sindbis virus 09_M_358/Cx_torrentium/Sweden/2009	Sweden	2009	x	x
MK045246	Sindbis virus SAAR_18141/Cx_univittatus/South Africa/1976	South Africa	1976	x	x
MK045247	Sindbis virus AR18132/Cx_univittatus/South Africa/1974	South Africa	1974	x	x
MK045249	Sindbis virus 4251SM6/Mosquito/Cameroon/1969	Cameroon	1969	x	x
MK045250	Sindbis virus SAAR_6071/Cx_univittatus/South Africa/1964	South Africa	1964	x	x
MK045251	Sindbis virus SK2_SM7V1/Cricetus/SlovakRepublic/1972	Slovakia	1972	x	x
MK045252	Sindbis virus AZ_16/Nycticorax_nycticorax/Russia/1977	Russia	1977	x	x
MK045253	Sindbis virus GREIS/Hyalomma_marginatum/Italy/1975	Italy	1975	x	x
MK045254	Sindbis virus M1855/Culex_molestus/Israel/1967	Israel	1967	x	x
MK045255	Sindbis virus R2_SM7V1/Rana_ridibunda/SlovakRepublic/1972	Slovakia	1972	x	x
MK045256	Sindbis virus 1038/Streptopelia_turtur/Israel/1964	Israel	1964	x	x
MK045257	Sindbis virus SA80_480/Cx_univittatus/Saudi/1980	Saudi Arabia	1980	x	x
MK045258	Sindbis virus SA80_394/Cx_univittatus/Saudi/1980	Saudi Arabia	1980	x	x
MK440626	Sindbis virus OTU14	Sweden	2009	x	x
MN389434	Sindbis virus P1_Mekrijarvi_2018	Finland	2018	x	x
MN389435	Sindbis virus FIN_2018_H_37	Finland	2018	x	x
MT121982	Sindbis virus AR-339	Egypt	1952	x	x
MT270144	Sindbis virus FIN_2018_H_25	Finland	2018	x	x
MT270145	Sindbis virus FIN_2018_H_05	Finland	2018	x	x

NC_001547	Sindbis virus			x	x
NC_016961	Whataroa virus				x
OK644705	Sindbis virus P29 Algeria	Algeria	2017	x	x
OL943983	Sindbis virus GAU14MP070/SA	South Africa	2014	x	x
OL943984	Sindbis virus KYA14MP133/SA	South Africa	2014	x	x
OL943985	Sindbis virus MAR14MP222/SA	South Africa	2014	x	x
OL943986	Sindbis virus KYA14MP134/SA	South Africa	2014	x	x
OM179803	Sindbis virus K512	Kenya	1971	x	x
ON003414	Sindbis virus LMP_B	Brazil	2021		x
ON003415	Sindbis virus LMP_A	Brazil	2021		x
OR183436	Sindbis virus MP762-UG-2019	Uganda	2019	x	x
OR882082	Sindbis virus TR339	USA	2001	x	x
PP496991	Sindbis virus 67 Banyo		2022		x
PP879145	Sindbis virus CA_JA220236_2022	Spain	2022		x
PP879146	Sindbis virus CA_JA220241_2022	Spain	2022		x
PP879147	Sindbis virus CA_JA220432_2022	Spain	2022		x
PP879148	Sindbis virus CA_JA22545_2022	Spain	2022		x
PP879149	Sindbis virus HU_22C1470_2022	Spain	2022		x
PP879150	Sindbis virus HU_HP597_2022	Spain	2022		x
PP879151	Sindbis virus SE_22C1621_2022	Spain	2022		x
PP879152	Sindbis virus SE_22C1639_2022	Spain	2022		x
PP879153	Sindbis virus SE_22C1668_2022	Spain	2022		x
PP879154	Sindbis virus SE_22C1689_2022	Spain	2022		x
PP879155	Sindbis virus SE_22C1706_2022	Spain	2022		x
PP879156	Sindbis virus SE_22C1774_2022	Spain	2022		x
PP879157	Sindbis virus SE_JA220058_2022	Spain	2022		x
PP879158	Sindbis virus SE_JA220061_2022	Spain	2022		x
PP879159	Sindbis virus SE_JA220120_2022	Spain	2022		x
PP879160	Sindbis virus SE_JA220877_2022	Spain	2022		x
U38304	Sindbis-like virus Girdwood S.A.			x	x

U38305	Sindbis-like virus S.A.AR86	South Africa	x	x

Supplementary Table S2. Sequence primers used for SINV tiling PCR amplification.

Primer Name	Primer pool	Sequence
SINV_0_LEFT	1	AGAAGCCAGTAGTHAACGTRGACG
SINV_0_RIGHT	1	GCGTTCCAGGTGGGCGATA
SINV_2_LEFT	1	CTGTGCTGAAGAAYGCYAAACT
SINV_2_RIGHT	1	ACTGRCARCCGGTAAGTACGAT
SINV_4_LEFT	1	TCCCCRYAAGAGAATCGAATGGA
SINV_4_RIGHT	1	CTTTCYGGCGTCATRGCATAC
SINV_6_LEFT	1	YAGACGCAGGAGCAGGAGGA
SINV_6_RIGHT	1	GCRCACCTCTCAGCCATTTCTTT
SINV_8_LEFT	1	GACCACCCYGTRCTATCAAAG
SINV_8_RIGHT	1	CCACTTCGTYTGRTCGCTCTT
SINV_10_LEFT	1	CTGAAACRTTCACCRAGACCATG
SINV_10_RIGHT	1	GCAAACAGCCAACTCCAYGAWGTT
SINV_1_LEFT	2	TGCAGAGCTGGCAYCTYCCAT
SINV_1_RIGHT	2	ACRTACTCTGTTTCTGCRAGCT
SINV_3_LEFT	2	TGTACGCRATCACATCAGAGCA
SINV_3_RIGHT	2	RTAGAYGGCACGGCAGACTCC
SINV_5_LEFT	2	TCTGTCGCCATYCCAYTGCTAT
SINV_5_RIGHT	2	TRTTGGCTTCRGTGGGCATCA
SINV_7_LEFT	2	GGAYCAACCRCTACTCGACTTGA
SINV_7_RIGHT	2	TGCWGCRGACCACTCTTCTGT
SINV_9_LEFT	2	CCRAAATTCGTGGGACGGGAAA
SINV_9_RIGHT	2	AACAYTGYGCTCCTCCCCACA
SINV_10_RIGHT_EXT		GCATTATGCACCATGCTTCCTCA
